# Cost-effectiveness of strengthening blood pressure classification in South Korea: comparing the 2017 ACC/AHA and KSH guidelines

**DOI:** 10.1186/s40885-024-00289-2

**Published:** 2024-11-01

**Authors:** KyungYi Kim, Min Ji Hong, Bomgyeol Kim, Hae-Young Lee, Tae Hyun Kim

**Affiliations:** 1 Department of Healthcare Management, Graduate School of Public Health, Yonsei University, 50-1, Yonsei-ro, Seodaemun-gu, Seoul, 03722 Republic of Korea; 2 Department of Medical Device Engineering and Management, Yonsei University Graduate School, Seoul, Republic of Korea; 3 Department of Public Health, Graduate School, Yonsei University, Seoul, Republic of Korea; 4 Department of Internal Medicine, Seoul National University College of Medicine, Seoul, Republic of Korea

**Keywords:** Economic evaluation, Cost-effectiveness analysis, Hypertension, Blood pressure, Cardiovascular diseases, Kidney failure, chronic

## Abstract

**Background:**

Hypertension is a significant risk factor for cardiovascular disease (CVD), with hypertension-related deaths increasing annually. While South Korea uses the Korean Society of Hypertension (KSH) guideline of 140/90 mmHg, the American College of Cardiology (ACC) and American Heart Association (AHA) updated their guidelines in 2017 to 130/80 mmHg. This study evaluates the cost-effectiveness of transitioning to the 2017 ACC/AHA guidelines by estimating early treatment impacts and potential CVD risk reduction.

**Methods:**

A Markov state-transition simulation model with a 10-year horizon was used to estimate cost-effectiveness, focusing on strengthening target blood pressure. Quality-adjusted life years (QALYs) served as the measure of effectiveness. Cohorts of 10,000 individuals representing South Koreans in their 20s through 80s were compared in scenario analyses from the healthcare system perspective. A 4.5% annual discount rate was applied to costs and effectiveness. Primary outcomes were incremental cost-effectiveness ratio (ICER) and net monetary benefit (NMB). The willingness-to-pay (WTP) threshold was < KRW 30,000,000/QALY gained. Probabilistic sensitivity analyses (PSAs) addressed model input parameter uncertainties.

**Results:**

The base-case analysis showed an ICER value of KRW 1,328,395/QALY gained across all populations. ICER values increased with age, from − KRW 3,138,071/QALY for 20-year-olds to KRW 16,613,013/QALY for individuals over 80. The 60s age group showed the greatest benefit with an incremental QALY gain of 0.46. All scenarios had ICERs below the WTP threshold, with a PSA probability of 98.0% that strengthening blood pressure classification could be cost-effective.

**Conclusions:**

This economic evaluation found that adopting the 2017 ACC/AHA guidelines may result in early treatment, reduce the potential incidence of CVD events, and be cost-effective across all age groups. The study findings have implications for policymakers deciding whether and when to revise official guidelines regarding target blood pressure levels, considering the impacts on public health and budgetary concerns.

**Supplementary Information:**

The online version contains supplementary material available at 10.1186/s40885-024-00289-2.

## Background

High blood pressure is associated with the incidence of cardiovascular disease (CVD) [1–3], making effective management and prevention of high blood pressure crucial. Previous studies have demonstrated that lowering blood pressure effectively reduces the risk of developing various CVDs and decreases mortality rates [4]. A decrease of 10 mmHg in systolic blood pressure (SBP) was associated with a reduction in the risk of major CVD events, coronary heart disease, stroke, and heart failure (HF). This reduction was correlated with a significant decline of 13% in all-cause mortality in the studied population [5].

To manage blood pressure from a public health perspective, each country establishes criteria for blood pressure, which are the standard treatment and target blood pressure. In the United States, the Eighth Joint National Committee (JNC-8) guideline established an SBP treatment threshold of 140 mmHg or higher and a diastolic blood pressure (DBP) threshold of 90 mmHg or higher for hypertension. The treatment goals were SBP of < 140 mmHg and DBP of < 90 mmHg. Europe and South Korea use the same criteria for hypertension diagnosis and treatment goals [6].

However, in 2017, the American College of Cardiology (ACC) and American Heart Association (AHA) updated their hypertension guidelines to reduce the target blood pressure from 140/90 mmHg to 130/80 mmHg. This change aimed to facilitate the early treatment of hypertension and more effectively prevent CVD [7]. In South Korea, the diagnostic and treatment criterion for hypertension remains at 140/90 mmHg, and lowering the target blood pressure to 130/80 mmHg is recommended only for high-risk groups or those with CVD [8]. Europe also maintains the diagnostic criterion for hypertension at 140/90 mmHg [9].

Lowering the diagnostic and treatment criteria for hypertension has the advantage of allowing early treatment of high blood pressure and prevention of CVD. However, it may lead to an increase in the hypertensive population and greater utilization of medical resources for treatment [10]. In the United States, approximately 31 million (13.7%) of the total population with a blood pressure of 130–139/80–89 mmHg are newly classified as hypertensive, resulting in a significant increase in the prevalence of hypertension in the United States from 31.9 to 45.6% [11].

In South Korea, according to the KSH guidelines, the population with hypertension is 4,084,994 (13.3%). However, using the 2017 ACC/AHA guidelines, an additional 8,805,621 people (30.4%) would be classified as hypertensive. In this case, 56.7% of the population aged 20 or older would be considered hypertensive [12]. As the number of patients with high blood increases, both personal and social medical costs increase. Therefore, it is essential to evaluate whether the long-term prevention of this condition is economically efficient compared to escalating costs. This assessment can be conducted through cost-effectiveness analysis.

This study compared the 2017 ACC/AHA hypertension guidelines, which have lower blood pressure thresholds for diagnosis and treatment, with the existing hypertension standards in South Korea, where higher thresholds are maintained. Based on the findings of previous studies, we chose coronary artery disease (CAD), stroke, HF, and chronic kidney disease (CKD) as significant complications of hypertension [1, 12–14] and analyzed data on their incidence and mortality rates.

Thus, this study aimed to assess the cost-effectiveness of adopting the 2017 ACC/AHA guidelines domestically and to determine which age group would benefit the most from lowering the criteria. Ultimately, this study is expected to offer insights regarding altering the hypertension treatment criteria and establishing national policies for managing chronic diseases.

## Methods

### Study overview

In this study, a Markov model was constructed to evaluate the cost-effectiveness of transitioning from the KSH guidelines to the 2017 ACC/AHA guidelines with a focus on strengthening the target blood pressure. To measure the age-specific cost-effectiveness of the intervention, we created hypothetical cohorts, each consisting of 10,000 South Koreans based on the population distribution (Supplemental Table 1). The base scenario represents all populations aged 20 to 99 years, while scenarios 1 to 7 represent the age groups of 20s, 30s, 40s, 50s, 60s, 70s, and over 80s, respectively. Quality-adjusted life years (QALYs) encompassing both quantity (life-years gained) and quality (health-related quality of life in utility value) estimates were used as effectiveness variables. According to the economic evaluation guidelines in South Korea [15], we adopted a healthcare system perspective that excludes non-medical (such as transportation and nursing care) and indirect costs (such as time costs), focusing solely on medical costs. The incremental cost-effectiveness ratio (ICER) and net monetary benefit (NMB) were calculated as primary outcomes to evaluate cost-effectiveness. Both QALYs and costs were discounted at a rate of 4.5% following the South Korean guidelines [15]. A willingness-to-pay (WTP) threshold was set at KRW 30,000,000 per QALY gained, reflecting the national norm. This study adhered to the Consolidated Health Economic Evaluation Reporting Standards (CHEERS) guidelines (Supplemental Table 2) [16].

### Model structure

**Fig. 1:**
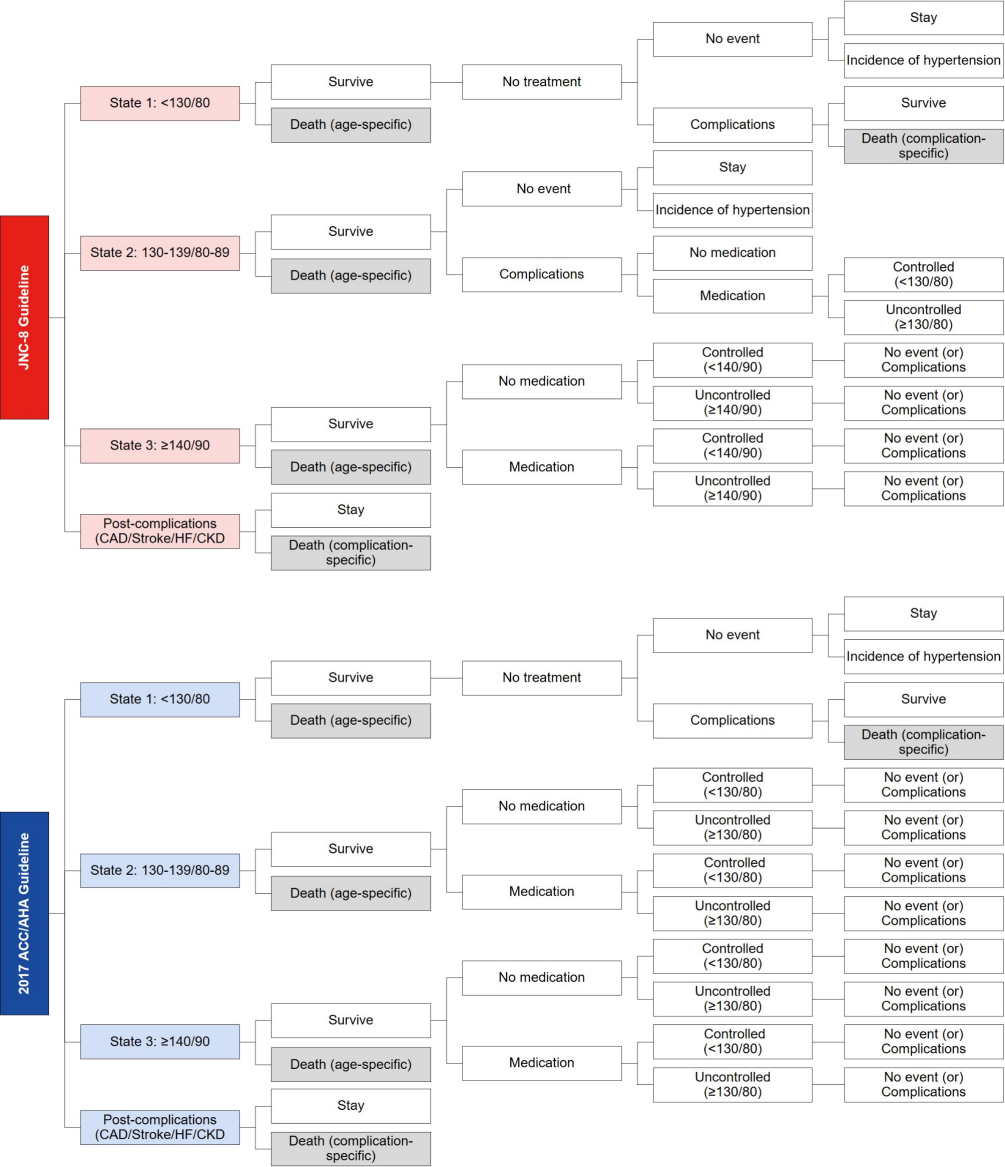
Markov structure

The analytical software program TreeAge Pro 2022 (TreeAge Software, Williamstown, MA, USA) was used to compare the cost-effectiveness of increasing the target blood pressure from the KSH guidelines to the 2017 ACC/AHA guidelines. The Markov model (Fig. 1) included eight health states: state 1 (< 130/80 mmHg), state 2 (130–139/80–89 mmHg), state 3 (≥ 140/90 mmHg), CAD, stroke, HF, CKD, and death. The differences between the KSH and 2017 ACC/AHA guidelines are as follows: (1) ‘In state 2, the KSH guidelines consider medication only for individuals with complications, while the 2017 ACC/AHA guidelines suggest medication for all individuals. (2) In state 3, the target blood pressure under the KSH guidelines is < 140/90 mmHg, whereas the 2017 ACC/AHA guidelines recommend an intensive target of < 130/80 mmHg. We hypothesized that intensive blood pressure control (< 130/80 mmHg) may reduce the incidence of complications. The 10-year Markov models were simulated with a one-year cycle length, and the detailed model structures are described in Supplemental Figs. 1 and 2. Due to the memoryless property of the Markov assumption, the model cannot reflect the patients’ previous health states [17]. The overall model structure was derived from previous studies [13, 18–25].

### Intervention and comparators

We compared the two guidelines to evaluate the optimal strategy. The control group followed the KSH guideline of 140/90 mmHg or higher for hypertensive blood pressure. The intervention group followed the 2017 ACC/AHA guideline of 130/80 mmHg or higher for high-risk individuals. We hypothesized that early diagnosis and treatment of high blood pressure might reduce the incidence of complications. We compared the incidence of complications according to blood pressure levels in different age groups and the corresponding death rates for both alternatives.

### Input variables

Table 1 presents an overview of the model input parameters used in this study. For the initial probabilities of health states, hazard ratios for the incidence of complications, and hazard ratios for the death rates, we utilized data from a previous study [12] that employed national-level health claim records from the National Health Insurance Service. The incidence of hypertension and complications (CAD, stroke, HF, and CKD), along with annual incremental costs of each health state, were obtained from a previous South Korean study that analyzed data from the Korea National Health and Nutrition Examination Survey [19]. Additional data were sourced from the Korean Hypertension Fact Sheet 2022 [26] to incorporate the treatment, medication, and control rates for each age group. The adjusted background mortality rates are presented in Supplemental Table 3. All costs were inflated to 2022, the most recent available data, in accordance with the Consumer Price Index [32]. Input parameters for scenarios 1 to 7 are presented in Supplemental Table 4.

**Table 1:** Input parameters for base case scenario representing all populations

	Value (Range)	Distribution	Source
**Transition Probabilities**			
**Initial Probabilities**			
State 1: <130/80 mmHg	0.432 (0.389–0.475)	beta	[12]
State 2: 130–139/80–89 mmHg	0.304 (0.274–0.334)	beta	[12]
State 3: ≥140/90 mmHg	0.264 (0.238–0.290)	beta	[12]
**State 1: <130/80 mmHg**			
Incidence of hypertension	0.0201 (0.0181–0.0221)	beta	[19]
Incidence of complications			
CAD	0.0010 (0.0009–0.0011)	beta	[19]
Stroke	0.0082 (0.0074–0.0091)	beta	[19]
HF	0.0018 (0.0016–0.0020)	beta	[19]
CKD	0.0005 (0.0004–0.0005)	beta	[19]
Death rate from			
CAD	0.0281 (0.0253–0.0309)	beta	[19]
Stroke	0.0233 (0.0210–0.0257)	beta	[19]
HF	0.0278 (0.0250–0.0305)	beta	[19]
CKD	0.0480 (0.0432–0.0528)	beta	[19]
HR for all-cause death (Ref: State 1)			
State 2: 130–139/80–89 mmHg	1.07 (0.97–1.18)	normal	[12]
State 3: ≥140/90 mmHg	1.36 (1.23–1.50)	normal	[12]
**State 2: 130–139/80–89 mmHg**			
Medication rate	0.541 (0.487–0.595)	beta	[26]
Control rate	0.740 (0.666–0.814)	beta	[26]
HR for incidence in State 2 vs. State 1			
CAD	1.30 (1.17–1.43)	normal	[12]
Stroke	1.36 (1.22–1.49)	normal	[12]
HF	1.24 (1.12–1.37)	normal	[12]
CKD	1.30 (1.17–1.43)	normal	Assumed^a^
HR for death rate in State 2 vs. State 1			
CAD, Stroke, HF, CKD	1.29 (1.16–1.42)	normal	[12]
**State 3: ≥140/90 mmHg**			
HR for incidence in State 3 vs. State 1			
CAD	1.79 (1.61–1.97)	normal	[12]
Stroke	2.32 (2.09–2.56)	normal	[12]
HF	1.98 (1.78–2.18)	normal	[12]
CKD	2.03 (1.83–2.23)	normal	Assumed^a^
HR for death rate in State 3 vs. State 1			
CAD, Stroke, HF, CKD	2.39 (2.15–2.63)	normal	[12]
**Death rate from post-complication states**			
Post-CAD / Post-Stroke / Post-HF / Post-CKD	0.0538 / 0.0736 / 0.0420 / 0.1340	beta	[27–30]
**Utilities**			
State 1 / State 2 / State 3	1.00 / 0.93 / 0.80	beta	[22]
Post-CAD / Post-Stroke / Post-HF / Post-CKD	0.70 / 0.65 / 0.73 / 0.78	beta	[22]
Acute-CAD / Acute-Stroke / Acute-HF / Acute-CKD	0.60 / 0.55 / 0.63 / 0.68	beta	[22]
**Costs (KRW)**			
**Annual cost**			
State 1: <130/80 mmHg	2,227,883 (2,005,095–2,450,672)	gamma	[19]
State 2: 130–139/80–89 mmHg	2,143,618 (1,929,257–2,357,980)	gamma	[19]
State 3: ≥140/90 mmHg	2,059,353 (1,853,418–2,265,289)	gamma	[19]
Post-CAD	4,793,441 (4,314,097–5,272,785)	gamma	[19]
Post-Stroke	5,180,983 (4,662,885–5,699,082)	gamma	[19]
Post-HF	3,745,489 (3,370,940–4,120,038)	gamma	[19]
Post-CKD	16,625,615 (14,963,053–18,288,176)	gamma	[19]
**Initial costs for acute complications**			
CAD	9,586,882 (8,628,194–10,545,571)	gamma	Assumed^b^
Stroke	10,361,967 (9,325,770–11,398,164)	gamma	Assumed^b^
HF	7,490,978 (6,741,880–8,240,076)	gamma	Assumed^b^
CKD	33,251,229 (29,926,106–36,576,352)	gamma	Assumed^b^
**Costs for medication therapy**			
Standard medication (target: <140/90 mmHg)	292,166 (262,949–321,382)	gamma	[31]
Intensive medication (target: <130/80 mmHg)	640,122 (576,110–704,134)	gamma	[31]

CAD, coronary artery disease; HF, heart failure; CKD, chronic kidney disease.

^a^ The hazard ratios for the incidence in states 2 and 3 compared with state 1 of CKD were assumed to be the average of other complications, including CAD, stroke, and HF.

^b^ The initial costs for acute complications were assumed to be double the annual costs.

### Statistical analyses

In the base-case analysis, we evaluated the cost-effectiveness of both strategies using ICER and NMB, applying an annual discount rate of 4.5% to both the effectiveness and cost variables. The ICER was calculated by dividing the incremental costs by the incremental effectiveness (QALY). If the ICER was lower than the WTP threshold, the results were considered cost-effective. The NMB was calculated as follows: NMB = (WTP) × (effectiveness) – (cost). When the NMB value was higher for a particular strategy, it was interpreted as being more cost-effective than the alternatives. Because we conducted scenario analyses, the ICER and NMB results could be compared across different age groups (20s–>80s) to determine which age group exhibited better cost-effectiveness. For both QALYs and costs, a half-cycle correction was applied to address the inaccuracies at the start or end of each cycle. To minimize the uncertainty associated with the nature of the economic evaluation, we performed a probabilistic sensitivity analysis (PSA) using the distributions listed in Table 1. A Monte Carlo simulation with 10,000 iterations for each scenario was conducted for the PSA, and variables used in the analysis were randomly sampled to determine the percentage of instances in which certain strategies were more optimal. The results are presented as incremental cost-effectiveness (ICE) scatter plots.

## Results

### Base-case analysis

**Fig. 2:**
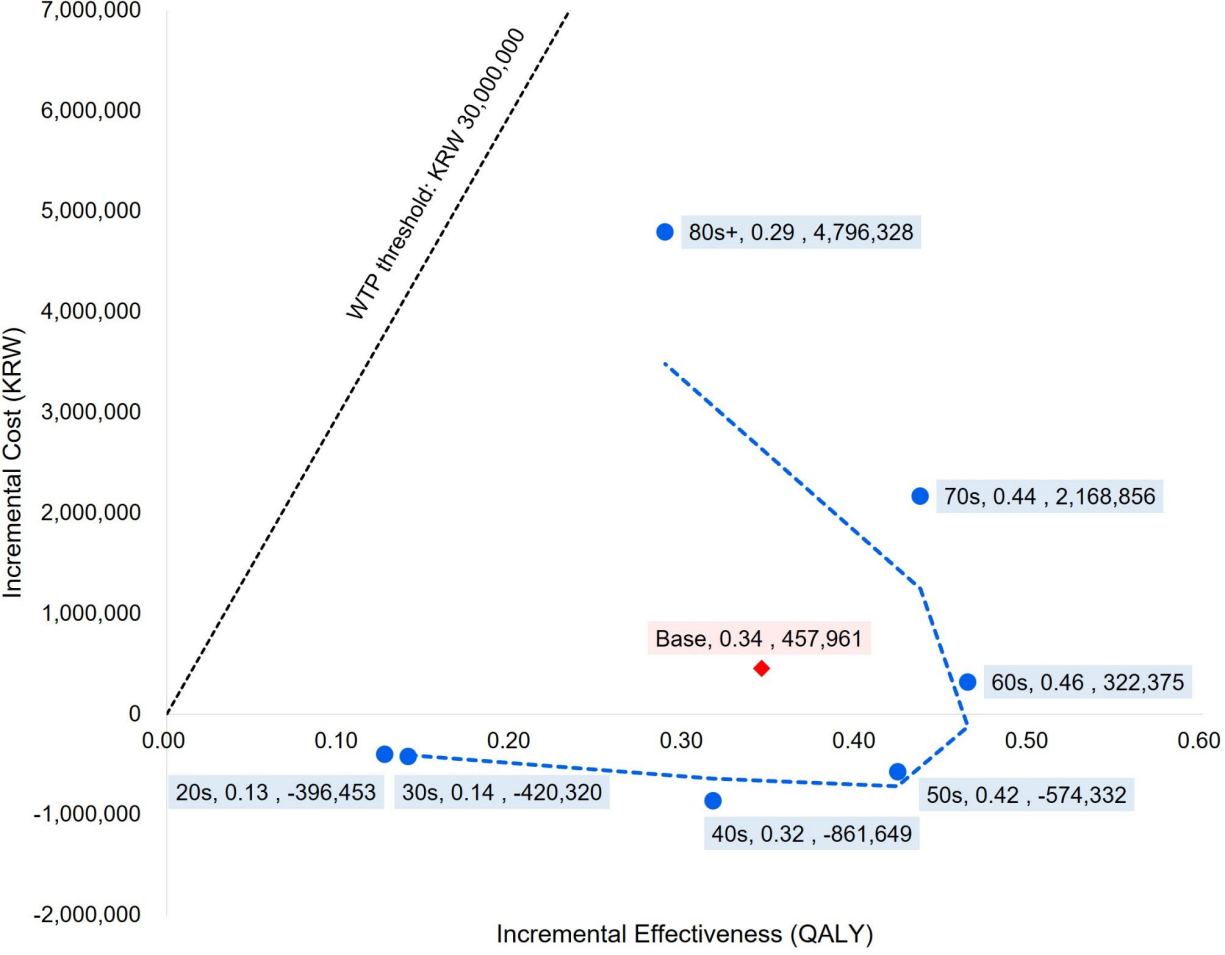
Base case results of all scenarios

Table 2 presents the results of the base case analysis. The ICER for the base scenario was KRW 1,328,395 per QALY gained with a base discount rate of 4.5%, which was below the WTP threshold. For scenarios 1–7, the ICER values increased from − KRW 3,138,071 to KRW 16,613,013 with ages ranging from individuals in their 20s to those over 80s. All populations showed cost-effectiveness; however, younger individuals demonstrated better cost-effectiveness. Furthermore, the NMBs of the intervention (2017 ACC/AHA guidelines) group were determined to be KRW 284,754,501 in scenario 1 (aged 20s) and KRW 143,822,676 in scenario 7 (aged over 80s), indicating that the younger age group doubled the monetary value compared to the older group. Figure 2 shows the incremental effectiveness and cost values for each scenario. All scenarios were below the WTP threshold of KRW 30,000,000, implying that the entire population was cost-effective when adjusting for the new hypertension criteria. Especially for scenarios 1–4 (aged 20–50 s), the incremental costs were negative, even with positive incremental effectiveness values. This indicates that younger populations aged < 60 years showed dominance with superior clinical effectiveness and cost savings. When considering the clinical effectiveness perspective only, scenario 5, with individuals aged 60s, was shown to be optimal with an incremental QALY of 0.46.

**Table 2:** Base case results of each scenario

Scenario	Strategy	Cost	Effectiveness	ICER	NMB
Base	Baseline	23,012,812	9.07		249,203,346
	Comparator	23,470,773	9.42	1,328,395	259,087,814
1 (age 20s)	Baseline	6,557,761	9.57		280,567,948
	Comparator	6,161,307	9.70	(3,138,071)	284,754,501
2 (age 30s)	Baseline	8,615,662	9.50		276,404,361
	Comparator	8,195,343	9.64	(3,005,447)	281,020,258
3 (age 40s)	Baseline	11,123,771	9.30		267,805,928
	Comparator	10,262,121	9.61	(2,722,752)	278,161,458
4 (age 50s)	Baseline	16,722,035	8.93		251,155,843
	Comparator	16,147,703	9.35	(1,356,392)	264,432,959
5 (age 60s)	Baseline	24,365,022	8.59		233,262,949
	Comparator	24,687,397	9.05	694,702	246,862,011
6 (age 70s)	Baseline	32,691,564	7.59		195,057,229
	Comparator	34,860,420	8.03	4,967,998	205,985,333
7 (age 80s+)	Baseline	30,355,834	5.68		139,957,731
	Comparator	35,152,161	5.97	16,613,013	143,822,676

Units for cost and effectiveness values are KRW and QALY, respectively.

ICER, incremental cost-effectiveness ratio; NMB, net monetary benefit; QALY, quality-adjusted life years; baseline, hypertension criteria based on the KSH guidelines; comparator, hypertension criteria based on the 2017 ACC/AHA guidelines.

### Sensitivity analysis

**Fig. 3:**
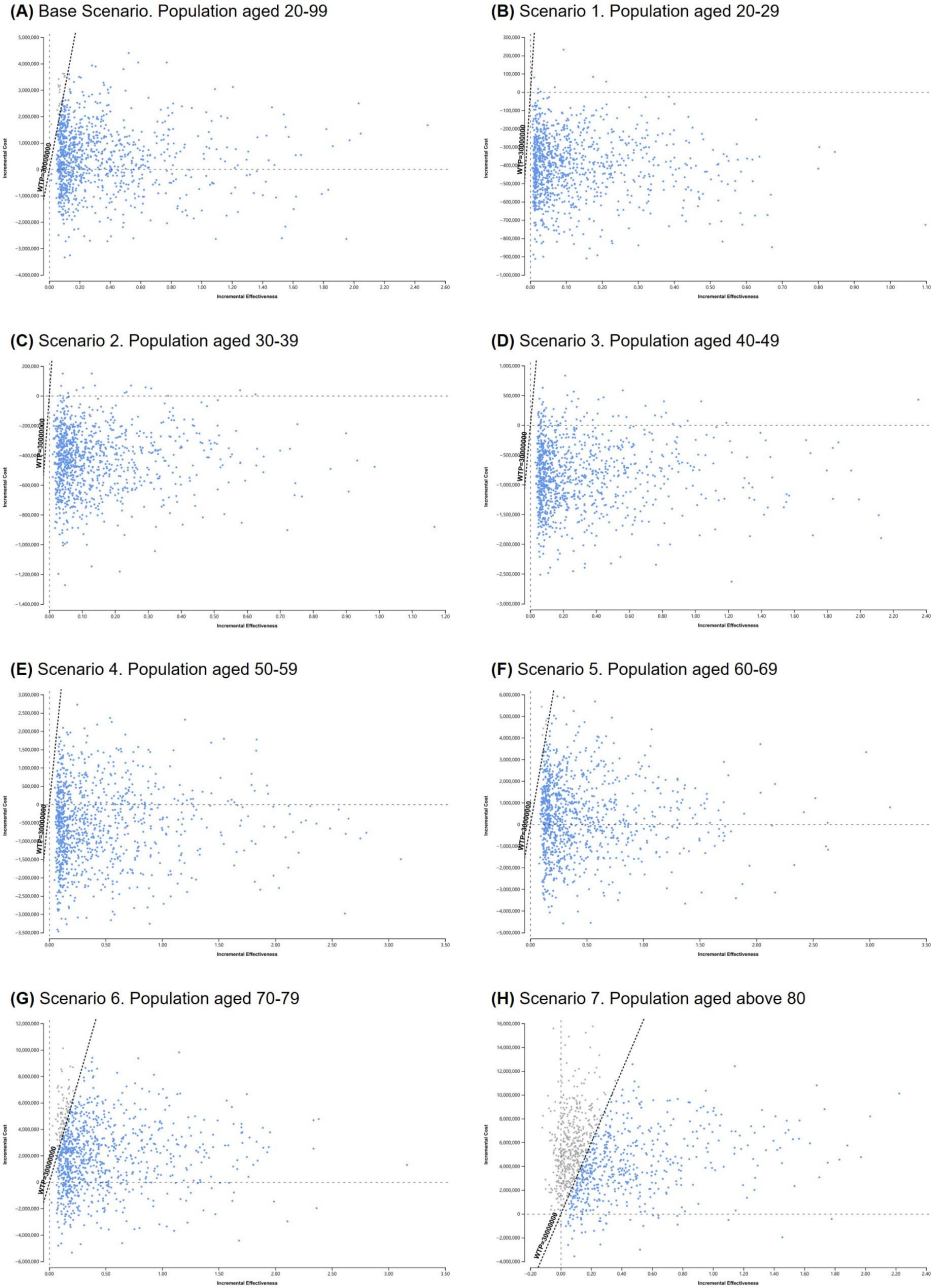
Results of the incremental cost-effectiveness scatterplot as probabilistic sensitivity analyses

A PSA was conducted to assess the percentage of a certain strategy being cost-effective when the input values differed from the base-case values. Figure 3 shows the ICE scatterplot of 10,000 iterations of the Monte Carlo simulation for each scenario. With a probability of 98.0%, transitioning the hypertension criteria from the KSH guidelines to the 2017 ACC/AHA guidelines was the most cost-effective strategy in the base scenario. For scenarios 1 to 7, the probabilities of the intervention group being the optimal strategy were found to be 100.0%, 100.0%, 100.0%, 99.9%, 99.3%, 90.8%, and 55.0%, respectively. Excluding individuals aged > 80 years, the strengthened blood pressure guidelines were cost-effective for more than 90% of all age groups.

## Discussion

### Study methods

In this study, we analyzed the cost-effectiveness of lowering the target blood pressure levels by introducing the 2017 AHA/ACC guidelines in South Korea. We conducted scenario analyses for individuals aged from their 20s to over 80s to examine age-specific differences in cost-effectiveness when adopting the strengthened blood pressure criteria. We incorporated age-specific values, including the incidence of complications, death rates from complications, hazard ratios, medication rates, control rates, and annual costs. Additionally, we utilized national-level claims data as input parameters to simulate the Markov model, aligning it closely with the local environmental settings. The Markov structure was designed by considering the major complications (including CAD, stroke, HF, and CKD) that can arise from hypertension, enabling us to anticipate the potential impacts in real-world situations. Furthermore, the WTP threshold in this study was KRW 30,000,000, which is lower than the gross domestic product (GDP) per capita in 2022 (USD 32,255, equivalent to KRW 42,554,022 [33]). While it is common to use one to three times the GDP as the WTP threshold in global cost-effectiveness analyses [34], we employed the South Korean norm monetary value to conservatively assess the results. If the WTP threshold were set at 1-GDP or higher, the ICER and NMB would be higher, and the percentage of the intervention being cost-effective would increase. Previous studies have examined the cost-effectiveness of intensive care versus standard care [13, 20, 23] or analyzed clinical changes in the new hypertension guidelines [12]. To the best of our knowledge, this study represents the first economic evaluation of the cost-effectiveness of transitioning the blood pressure level from 140/90 mmHg to 130/80 mmHg by age group.

### Study results

The study results showed that it was predominantly cost-effective to implement new hypertension criteria based on the 2017 ACC/AHA guidelines, particularly for younger populations. It is necessary to establish age- and complication-specific policies to lower the target blood pressure. For individuals aged 20–30 s, we observed that medication rates among patients with prevalent hypertension were under 15%, the lowest among all age groups. While a previous study addressed low medication rates in younger age groups [35], further research is required to determine the most cost-effective strategy: lowering the target blood pressure level to 130/80 mmHg or maintaining the criteria at 140/90 mmHg and encouraging younger populations to increase medication rates. From the perspective of clinical effectiveness, individuals in their 60s showed the highest increase. Given the minimal incremental costs of adopting the new criteria in age groups from 20s to 60s and considering limited budgets, it might be beneficial to implement new policies for the 60s age group in advance.

### Limitations

This study had several limitations. First, due to the inherent uncertainty in economic evaluation studies, numerous assumptions were made during the analysis. To mitigate this, we conducted a sensitivity analysis (PSA). By randomly sampling the input parameter values and simulating 10,000 iterations for each scenario, we demonstrated the robustness of the results. Second, this study was conducted from a healthcare system perspective, excluding non-medical and indirect costs in accordance with the South Korean economic evaluation guidelines. Considering that the incidence of complications may lead to increased transportation, nursing, and time costs, the results from a societal perspective are anticipated to be more cost-effective than those from a healthcare system perspective. Third, the Markov structure in this study did not represent all populations. The risk of disease incidence can vary based on demographic characteristics (e.g., sex, income level, marital status, education level, and employment) and individual lifestyle habits (e.g., drinking, smoking, and exercise). Fourth, the two guidelines used in this study may not significantly differ from each other as they may be interpreted differently in real-world clinical settings. We hypothesized that all individuals in State 2 are treated according to the 2017 ACC/AHA guidelines, while only those with complications are treated according to the JNC-8 guidelines. Additionally, there might be complications associated with hypertension beyond CAD, HF, stroke, and CKD, such as hypotension or electrolyte abnormalities. Although not all variables were accounted for, the analysis focused on the major complications of hypertension and age-specific risks.

## Conclusions

In conclusion, strengthening the target blood pressure level from the KSH guidelines to the 2017 ACC/AHA guidelines has resulted in the early treatment of the potential incidence of complications and has proven to be cost-effective across all age groups. These findings have implications for policymakers deciding whether and when to strengthen the target blood pressure level based on public health and cost-effectiveness.

## Electronic supplementary material

Below is the link to the electronic supplementary material.

Supplementary Material 1

## Data Availability

Not applicable.

## Abbreviations

CAD: coronary artery disease
CEA: cost-effectiveness analysis
CKD: chronic kidney disease
CVD: cardiovascular disease
DBP: diastolic blood pressure
HF: heart failure
GDP: gross domestic product
ICER: incremental cost-effectiveness ratio
NMB: net monetary benefit
PSA: probabilistic sensitivity analysis
QALY: quality-adjusted life years
SBP: systolic blood pressure
WTP: willingness-to-pay
