# Supplementary material for: Cost-effectiveness of strengthening blood pressure classification in South Korea: comparing the 2017 ACC/AHA and KSH guidelines
Source: Clin Hypertens. 2024 Nov 1;30:34. doi: 10.1186/s40885-024-00289-2 (PMC11528990; doi:10.1186/s40885-024-00289-2)
Supplement: Supplementary file 1 — Supplementary Material 1 [file 40885_2024_289_MOESM1_ESM.docx]

**Cost-effectiveness of strengthening blood pressure classification in South Korea: Comparing the 2017 ACC/AHA and KSH guidelines**

**Supplementary Tables**

**Table 1.** Population distribution by age

**Table 2.** Consolidated Health Economic Evaluation Reporting Standards 2022 (CHEERS 2022) checklist.

**Table 3.** Background mortality of each Markov cycle

**Table 4.** Input parameters for each scenario

**Supplementary Figures**

**Figure 1.** Markov structure for baseline (KSH guideline)

**Figure 2.** Markov structure for comparator (2017 ACC/AHA guideline)

Supplementary Table 1. Population distribution by age

| **Cycle** | **Population** | **Cycle** | **Population** | **Cycle** | **Population** |
| --- | --- | --- | --- | --- | --- |
| 0 | 411,089 | 34 | 796,086 | 68 | 438,903 |
| 1 | 435,097 | 35 | 802,347 | 69 | 434,793 |
| 2 | 431,594 | 36 | 820,357 | 70 | 329,832 |
| 3 | 438,394 | 37 | 776,971 | 71 | 326,774 |
| 4 | 476,429 | 38 | 713,342 | 72 | 333,374 |
| 5 | 469,329 | 39 | 738,990 | 73 | 344,726 |
| 6 | 450,309 | 40 | 727,930 | 74 | 401,588 |
| 7 | 436,023 | 41 | 766,561 | 75 | 324,161 |
| 8 | 460,205 | 42 | 837,974 | 76 | 296,267 |
| 9 | 471,257 | 43 | 848,890 | 77 | 294,346 |
| 10 | 432,275 | 44 | 850,444 | 78 | 262,995 |
| 11 | 426,227 | 45 | 906,302 | 79 | 240,711 |
| 12 | 465,254 | 46 | 875,408 | 80 | 218,580 |
| 13 | 474,608 | 47 | 888,357 | 81 | 206,564 |
| 14 | 490,248 | 48 | 886,109 | 82 | 175,637 |
| 15 | 561,090 | 49 | 813,427 | 83 | 148,380 |
| 16 | 617,413 | 50 | 797,783 | 84 | 131,538 |
| 17 | 600,605 | 51 | 818,517 | 85 | 102,324 |
| 18 | 627,134 | 52 | 768,463 | 86 | 95,767 |
| 19 | 650,486 | 53 | 808,561 | 87 | 81,094 |
| 20 | 668,404 | 54 | 837,240 | 88 | 66,418 |
| 21 | 674,881 | 55 | 902,996 | 89 | 53,224 |
| 22 | 682,729 | 56 | 889,333 | 90 | 37,297 |
| 23 | 683,943 | 57 | 797,473 | 91 | 31,676 |
| 24 | 690,677 | 58 | 751,771 | 92 | 22,681 |
| 25 | 651,092 | 59 | 734,477 | 93 | 22,411 |
| 26 | 621,314 | 60 | 677,011 | 94 | 15,945 |
| 27 | 605,245 | 61 | 688,651 | 95 | 10,371 |
| 28 | 593,201 | 62 | 581,525 | 96 | 7,261 |
| 29 | 598,118 | 63 | 508,744 | 97 | 4,802 |
| 30 | 610,920 | 64 | 554,455 | 98 | 3,196 |
| 31 | 622,398 | 65 | 411,138 | 99 | 2,168 |
| 32 | 657,551 | 66 | 443,962 |  |  |
| 33 | 728,644 | 67 | 450,728 |  |  |

Supplementary Table 2. Consolidated Health Economic Evaluation Reporting Standards 2022 (CHEERS 2022) checklist

| **Topic** | **No.** | **Item** | **Location where item is reported** |
| --- | --- | --- | --- |
| **Title and abstract** |  |  |  |
| Title | 1 | Identify the study as an economic evaluation and specify the interventions being compared. | Title |
| Abstract | 2 | Provide a structured summary that highlights context, key methods, results, and alternative analyses. | Abstract |
| **Introduction** |  |  |  |
| Background and objectives | 3 | Give the context for the study, the study question, and its practical relevance for decision-making in policy or practice. | Introduction section |
| **Methods** |  |  |  |
| Health economic analysis plan | 4 | Indicate whether a health economic analysis plan was developed and where available. | Not reported |
| Study population | 5 | Describe characteristics of the study population (such as age range, demographics, socioeconomic, or clinical characteristics). | Methods (study overview) section |
| Setting and location | 6 | Provide relevant contextual information that may influence findings. | Methods (study overview) section |
| Comparators | 7 | Describe the interventions or strategies being compared and why chosen. | Methods (intervention and comparators) section |
| Perspective | 8 | State the perspective(s) adopted by the study and why chosen. | Methods (study overview) section |
| Time horizon | 9 | State the time horizon for the study and why appropriate. | Methods (study overview) section |
| Discount rate | 10 | Report the discount rate(s) and reason chosen. | Methods (study overview) section |
| Selection of outcomes | 11 | Describe what outcomes were used as the measure(s) of benefit(s) and harm(s). | Methods (study overview) section |
| Measurement of outcomes | 12 | Describe how outcomes used to capture benefit(s) and harm(s) were measured. | Methods (input variables) section |
| Valuation of outcomes | 13 | Describe the population and methods used to measure and value outcomes. | Methods (model structure) section |
| Measurement and valuation of resources and costs | 14 | Describe how costs were valued. | Methods (input variables) section |
| Currency, price date, and conversion | 15 | Report the dates of the estimated resource quantities and unit costs, plus the currency and year of conversion. | Methods (input variables) section |
| Rationale and description of model | 16 | If modeling is used, describe in detail and why used. Report if the model is publicly available and where it can be assessed. | Methods (model structure) section |
| Analytics and assumptions | 17 | Describe any methods for analyzing or statistically transforming data, any extrapolation methods, and approaches for validating and model used. | Methods (study overview) section |
| Characterizing heterogeneity | 18 | Describe any methods used for estimating how the results of the study vary for subgroups. | Methods (statistical analyses) section |
| Characterizing distributional effects | 19 | Describe how impacts are distributed across different individuals or adjustments made to reflect priority populations. | Methods (statistical analyses) section |
| Characterizing uncertainty | 20 | Describe methods to characterize any sources of uncertainty in the analysis. | Methods (statistical analyses) section |
| Approach to engagement with patients and others affected by the study | 21 | Describe any approaches to engage patients or service recipients, the general public, communities, or stakeholders (e.g., clinicians or payers) in the design of the study. | Not reported |
| **Results** |  |  |  |
| Study parameters | 22 | Report all analytic inputs (e.g., values, ranges, references) including uncertainty or distributional assumptions. | Methods (input variables) section, Table 1 |
| Summary of main results | 23 | Report the mean values for the main categories of costs and outcomes of interest and summarize them in the most appropriate overall measure. | Results section, Table 2, Figure 2 |
| Effect of uncertainty | 24 | Describe how uncertainty about analytic judgments, inputs, or projections affects findings. Report the effect of the choice of discount rate and time horizon, if applicable. | Results section, Figure 3 |
| Effect of engagement with patients and others affected by the study | 25 | Report any difference in patient/service recipient, general public, community, or stakeholder involvement made to the approach or findings of the study. | Results section, Figure 3 |
| **Discussion** |  |  |  |
| Study findings, limitations, generalizability, and current knowledge | 26 | Report key findings, limitations, ethical, or equity considerations not captured and how these could impact patients, policy, or practice. | Discussion section |
| **Other relevant information** |  |  |  |
| Source of funding | 27 | Describe how the study was funded and any role of the funder in the identification, design, conduct, and reporting of the analysis. | Funding declaration |
| Conflicts of interest | 28 | Report authors’ conflicts of interest according to journal or International Committee of Medical Journal Editors requirements. | Competing interest declaration |

Supplementary Table 3. Background mortality of each Markov cycle^2^

| **Cycle** | **Age-specific mortality** | **Cycle** | **Age-specific mortality** | **Cycle** | **Age-specific mortality** |
| --- | --- | --- | --- | --- | --- |
| 0 | 0.002333 | 34 | 0.000554 | 68 | 0.009330 |
| 1 | 0.000159 | 35 | 0.000602 | 69 | 0.008971 |
| 2 | 0.000093 | 36 | 0.000665 | 70 | 0.011004 |
| 3 | 0.000119 | 37 | 0.000795 | 71 | 0.012435 |
| 4 | 0.000100 | 38 | 0.000846 | 72 | 0.012957 |
| 5 | 0.000055 | 39 | 0.000875 | 73 | 0.015104 |
| 6 | 0.000075 | 40 | 0.000932 | 74 | 0.015723 |
| 7 | 0.000055 | 41 | 0.000982 | 75 | 0.017689 |
| 8 | 0.000077 | 42 | 0.000995 | 76 | 0.022710 |
| 9 | 0.000046 | 43 | 0.001206 | 77 | 0.024519 |
| 10 | 0.000066 | 44 | 0.001208 | 78 | 0.031681 |
| 11 | 0.000073 | 45 | 0.001304 | 79 | 0.030911 |
| 12 | 0.000109 | 46 | 0.001567 | 80 | 0.037045 |
| 13 | 0.000119 | 47 | 0.001682 | 81 | 0.045231 |
| 14 | 0.000147 | 48 | 0.001817 | 82 | 0.048356 |
| 15 | 0.000149 | 49 | 0.002032 | 83 | 0.055314 |
| 16 | 0.000205 | 50 | 0.002040 | 84 | 0.064307 |
| 17 | 0.000231 | 51 | 0.002250 | 85 | 0.073583 |
| 18 | 0.000267 | 52 | 0.002421 | 86 | 0.079999 |
| 19 | 0.000293 | 53 | 0.002493 | 87 | 0.091029 |
| 20 | 0.000304 | 54 | 0.002738 | 88 | 0.106232 |
| 21 | 0.000332 | 55 | 0.003188 | 89 | 0.114792 |
| 22 | 0.000328 | 56 | 0.003256 | 90 | 0.134210 |
| 23 | 0.000404 | 57 | 0.003501 | 91 | 0.145925 |
| 24 | 0.000387 | 58 | 0.003980 | 92 | 0.162783 |
| 25 | 0.000430 | 59 | 0.004060 | 93 | 0.178000 |
| 26 | 0.000443 | 60 | 0.004547 | 94 | 0.193871 |
| 27 | 0.000422 | 61 | 0.004601 | 95 | 0.238753 |
| 28 | 0.000450 | 62 | 0.005121 | 96 | 0.235394 |
| 29 | 0.000493 | 63 | 0.005655 | 97 | 0.283927 |
| 30 | 0.000483 | 64 | 0.005884 | 98 | 0.281869 |
| 31 | 0.000540 | 65 | 0.006804 | 99 | 0.299302 |
| 32 | 0.000519 | 66 | 0.007164 |  |  |
| 33 | 0.000564 | 67 | 0.007757 |  |  |

The background mortality used in this study was referred from the 2021 age-specific mortality rate in South Korea.

Supplementary Table 4. Input parameters for each scenario

|  | **Scenario 1** | **Scenario 2** | **Scenario 3** | **Scenario 4** | **Scenario 5** | **Scenario 6** | **Scenario 7** | **Source** |
| --- | --- | --- | --- | --- | --- | --- | --- | --- |
| **Transition Probabilities** |  |  |  |  |  |  |  |  |
| **Initial Probabilities** |  |  |  |  |  |  |  |  |
| State 1: <130/80mmHg | 0.580 | 0.580 | 0.473 | 0.311 | 0.311 | 0.193 | 0.146 | (12) |
| State 2: 130-139/80-89mmHg | 0.333 | 0.333 | 0.328 | 0.275 | 0.275 | 0.212 | 0.194 | (12) |
| State 3: ≥140/90mmHg | 0.087 | 0.087 | 0.199 | 0.414 | 0.414 | 0.595 | 0.660 | (12) |
| **State 1: <130/80mmHg** |  |  |  |  |  |  |  |  |
| Incidence of hypertension | 0.0029 | 0.0078 | 0.0191 | 0.0322 | 0.0414 | 0.0515 | 0.0449 | (19) |
| Incidence of complications |  |  |  |  |  |  |  |  |
| CAD | 0.0001 | 0.0002 | 0.0005 | 0.0010 | 0.0017 | 0.0023 | 0.0014 | (19) |
| Stroke | 0.0008 | 0.0014 | 0.0029 | 0.0065 | 0.0121 | 0.0213 | 0.0125 | (19) |
| HF | 0.0002 | 0.0003 | 0.0006 | 0.0013 | 0.0022 | 0.0044 | 0.0037 | (19) |
| CKD | 0.0001 | 0.0001 | 0.0002 | 0.0004 | 0.0006 | 0.0011 | 0.0006 | (19) |
| Death rate from |  |  |  |  |  |  |  |  |
| CAD | 0.0046 | 0.0000 | 0.0067 | 0.0103 | 0.0166 | 0.0462 | 0.1123 | (19) |
| Stroke | 0.0000 | 0.0044 | 0.0055 | 0.0068 | 0.0140 | 0.0374 | 0.0953 | (19) |
| HF | 0.0051 | 0.0020 | 0.0072 | 0.0089 | 0.0134 | 0.0419 | 0.1159 | (19) |
| CKD | 0.0089 | 0.0033 | 0.0103 | 0.0243 | 0.0421 | 0.0836 | 0.1636 | (19) |
| HR for all-cause death (Ref: State 1) |  |  |  |  |  |  |  |  |
| State 2: <140/90mmHg | 1.08 | 1.08 | 1.14 | 1.09 | 1.09 | 1.03 | 1.00 | (12) |
| State 3: ≥140/90mmHg | 1.53 | 1.53 | 1.54 | 1.33 | 1.33 | 1.19 | 1.10 | (12) |
| **State 2: 130-139/80-89mmHg** |  |  |  |  |  |  |  |  |
| Medication rate | 0.142 | 0.142 | 0.473 | 0.605 | 0.785 | 0.844 | 0.796 | (26) |
| Control rate | 0.774 | 0.774 | 0.753 | 0.738 | 0.772 | 0.683 | 0.685 | (26) |
| HR for incidence in State 2 vs State 1 |  |  |  |  |  |  |  |  |
| CAD | 1.48 | 1.48 | 1.44 | 1.30 | 1.30 | 1.14 | 0.95 | (12) |
| Stroke | 1.40 | 1.40 | 1.57 | 1.39 | 1.39 | 1.23 | 1.11 | (12) |
| HF | 1.40 | 1.40 | 1.29 | 1.18 | 1.18 | 1.10 | 1.14 | (12) |
| CKD | 1.43 | 1.43 | 1.43 | 1.29 | 1.29 | 1.16 | 1.07 | Assumed |
| HR for death rate in State 2 vs State 1 |  |  |  |  |  |  |  |  |
| CAD, Stroke, HF, CKD | 1.32 | 1.32 | 1.55 | 1.29 | 1.29 | 1.16 | 1.09 | (12) |
| **State 3: ≥140/90mmHg** |  |  |  |  |  |  |  |  |
| HR for incidence in State 3 vs State 1 |  |  |  |  |  |  |  |  |
| CAD | 2.43 | 2.43 | 1.97 | 1.53 | 1.53 | 1.40 | 1.24 | (12) |
| Stroke | 3.19 | 3.19 | 2.91 | 2.07 | 2.07 | 1.57 | 1.26 | (12) |
| HF | 3.04 | 3.04 | 2.14 | 1.58 | 1.58 | 1.26 | 1.22 | (12) |
| CKD | 2.89 | 2.89 | 2.34 | 1.73 | 1.73 | 1.41 | 1.24 | Assumed |
| HR for death rate in State 3 vs State 1 |  |  |  |  |  |  |  |  |
| CAD, Stroke, HF, CKD | 3.08 | 3.08 | 3.24 | 2.17 | 2.17 | 1.60 | 1.37 | (12) |
| **Death rate from post-complication states** |  |  |  |  |  |  |  |  |
| Post-CAD / Post-Stroke / Post-HF / Post-CKD |  |  |  |  |  |  |  | (27-30) |
| **Utilities** |  |  |  |  |  |  |  |  |
| State1 / State2 / State3 |  |  |  |  |  |  |  | (22) |
| Post-CAD / Post-Stroke / Post-HF / Post-CKD |  |  |  |  |  |  |  | (22) |
| Acute-CAD / Acute-Stroke / Acute-HF / Acute-CKD |  |  |  |  |  |  |  | (22) |
| **Costs** |  |  |  |  |  |  |  |  |
| **Annual cost** |  |  |  |  |  |  |  |  |
| State 1: <130/80mmHg | 476,890 | 677,707 | 873,463 | 1,438,934 | 2,391,570 | 3,893,329 | 5,843,291 | (19) |
| State 2: 130-139/80-89mmHg | 754,978 | 955,056 | 1,108,750 | 1,576,147 | 2,357,576 | 3,540,795 | 4,712,027 | (19) |
| State 3: ≥140/90mmHg | 1,033,066 | 1,232,405 | 1,344,037 | 1,713,360 | 2,323,582 | 3,188,262 | 3,580,763 | (19) |
| Post-CAD | 2,453,955 | 4,439,274 | 5,346,020 | 4,911,610 | 4,758,465 | 5,889,568 | 5,755,196 | (19) |
| Post-Stroke | 8,222,940 | 4,154,471 | 4,559,068 | 4,228,191 | 4,320,605 | 4,944,606 | 5,837,003 | (19) |
| Post-HF | 4,872,943 | 2,525,518 | 2,050,164 | 2,514,158 | 3,949,370 | 5,122,824 | 5,183,446 | (19) |
| Post-CKD | 13,412,787 | 17,226,631 | 18,435,144 | 18,037,349 | 18,471,124 | 16,552,633 | 14,243,636 | (19) |
| **Initial costs for acute complications** |  |  |  |  |  |  |  |  |
| CAD | 4,907,910 | 8,878,548 | 10,692,040 | 9,823,221 | 9,516,930 | 11,779,135 | 11,510,393 | Assumed |
| Stroke | 16,445,879 | 8,308,943 | 9,118,136 | 8,456,382 | 8,641,210 | 9,889,213 | 11,674,005 | Assumed |
| HF | 9,745,885 | 5,051,036 | 4,100,329 | 5,028,316 | 7,898,741 | 10,245,647 | 10,366,891 | Assumed |
| CKD | 26,825,573 | 34,453,262 | 36,870,288 | 36,074,698 | 36,942,248 | 33,105,265 | 28,487,272 | Assumed |
| **Costs for medication therapy** |  |  |  |  |  |  |  |  |
| Standard medication (target: <140/90mmHg) | 292,166 | 292,166 | 292,166 | 292,166 | 292,166 | 292,166 | 292,166 | (31) |
| Intensive medication (target: <130/80mmHg) | 640,122 | 640,122 | 640,122 | 640,122 | 640,122 | 640,122 | 640,122 | (31) |

Scenario 1: population aged between 20 to 29.

Scenario 2: population aged between 30 to 39.

Scenario 3: population aged between 40 to 49.

Scenario 4: population aged between 50 to 59.

Scenario 5: population aged between 60 to 69.

Scenario 6: population aged between 70 to 79.

Scenario 7: population aged above 80.


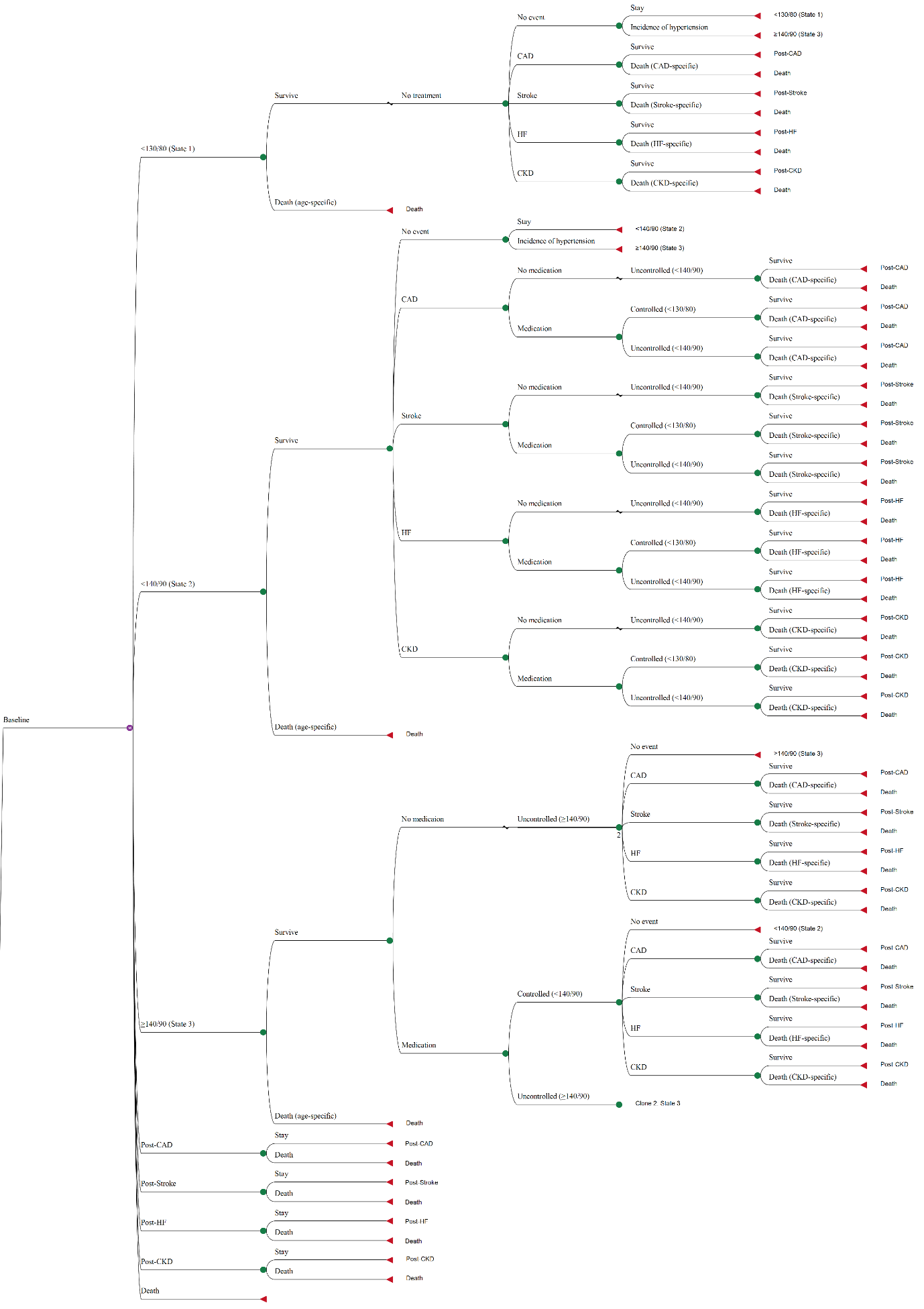


Supplementary Figure 1. Markov structure for baseline (KSH guideline)


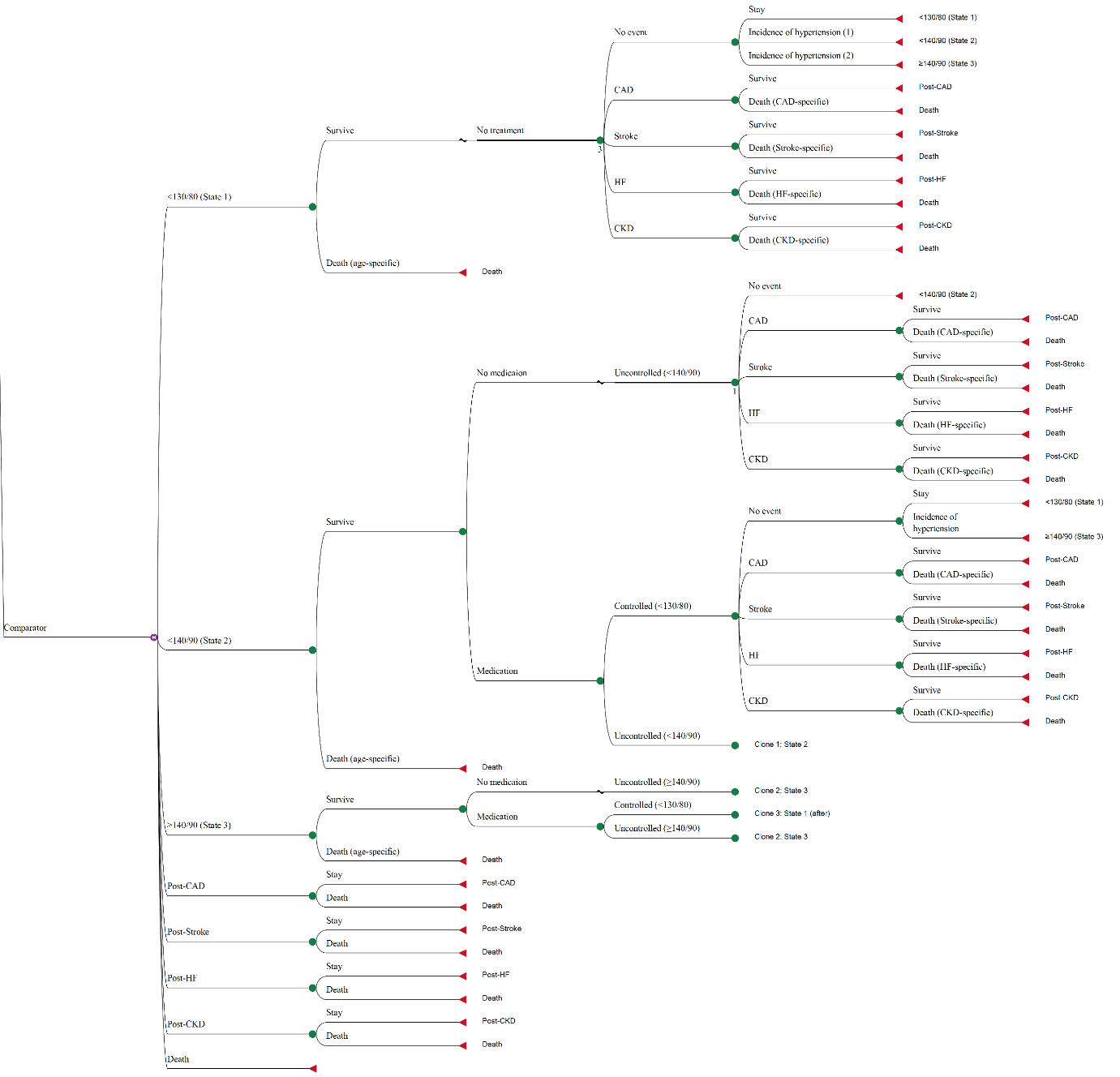


Supplementary Figure 2. Markov structure for comparator (2017 ACC/AHA guideline)
